# Supplementary material for: Stacking Model‐Based Classifiers for Dealing With Multiple Sets of Noisy Labels
Source: Biom J. 2025 Mar 12;67(2):e70042. doi: 10.1002/bimj.70042 (PMC11898607; doi:10.1002/bimj.70042)
Supplement: Supplementary file 1 — Supporting Information [file BIMJ-67-e70042-s001.zip › density-based-ensemble-model-main/competitors_real_data/netlab-master/netlab-master/help/mlp.htm]

Netlab Reference Manual mlp


# mlp

## Purpose

Create a 2-layer feedforward network.

## Synopsis

```
net = mlp(nin, nhidden, nout, func)
net = mlp(nin, nhidden, nout, func, prior)
net = mlp(nin, nhidden, nout, func, prior, beta)
```

## Description

`net = mlp(nin, nhidden, nout, func)` takes the number of inputs,
hidden units and output units for a 2-layer feed-forward network,
together with a string `func` which specifies the output unit
activation function, and returns a data structure `net`. The
weights are drawn from a zero mean, unit variance isotropic Gaussian,
with varianced scaled by the fan-in of the hidden or output units as
appropriate. This makes use of the Matlab function
`randn` and so the seed for the random weight initialization can be
set using `randn('state', s)` where `s` is the seed value.
The hidden units use the `tanh` activation function.

The fields in `net` are

```
  type = 'mlp'
  nin = number of inputs
  nhidden = number of hidden units
  nout = number of outputs
  nwts = total number of weights and biases
  actfn = string describing the output unit activation function:
      'linear'
      'logistic
      'softmax'
  w1 = first-layer weight matrix
  b1 = first-layer bias vector
  w2 = second-layer weight matrix
  b2 = second-layer bias vector
```

Here `w1` has dimensions `nin` times `nhidden`, `b1` has
dimensions `1` times `nhidden`, `w2` has
dimensions `nhidden` times `nout`, and `b2` has
dimensions `1` times `nout`.

`net = mlp(nin, nhidden, nout, func, prior)`, in which `prior` is
a scalar, allows the field `net.alpha` in the data structure
`net` to be set, corresponding to a zero-mean isotropic Gaussian
prior with inverse variance with value `prior`. Alternatively,
`prior` can consist of a data structure with fields `alpha`
and `index`, allowing individual Gaussian priors to be set over
groups of weights in the network. Here `alpha` is a column vector
in which each element corresponds to a separate group of weights,
which need not be mutually exclusive. The membership of the groups is
defined by the matrix `indx` in which the columns correspond to
the elements of `alpha`. Each column has one element for each
weight in the matrix, in the order defined by the function
`mlppak`, and each element is 1 or 0 according to whether the
weight is a member of the corresponding group or not. A utility
function `mlpprior` is provided to help in setting up the
`prior` data structure.

`net = mlp(nin, nhidden, nout, func, prior, beta)` also sets the
additional field `net.beta` in the data structure `net`, where
beta corresponds to the inverse noise variance.

## See Also

`mlpprior`, `mlppak`, `mlpunpak`, `mlpfwd`, `mlperr`, `mlpbkp`, `mlpgrad`

---

**Pages:**
Index


---

Copyright (c) Ian T Nabney (1996-9)
